# Generation gaps in US public opinion on renewable energy and climate change

**DOI:** 10.1371/journal.pone.0217608

**Published:** 2019-07-10

**Authors:** Lawrence C. Hamilton, Joel Hartter, Erin Bell

**Affiliations:** 1 Department of Sociology, University of New Hampshire, Durham, New Hampshire, United States of America; 2 Environmental Studies Program, University of Colorado, Boulder, Colorado, United States of America; 3 Civil and Environmental Engineering, University of New Hampshire, Durham, New Hampshire, United States of America; Mälardalen University, SWEDEN

## Abstract

The topics of climate change and renewable energy are often linked in policy discussions and scientific analysis, but public opinion on these topics exhibits both overlap and divergence. Although renewable energy has potentially broader acceptance than anthropogenic climate change, it can also face differently-based opposition. Analyses of US and regional surveys, including time series of repeated surveys in New Hampshire (2010–2018) and northeast Oregon (2011–2018), explore the social bases and trends of public views on both issues. Political divisions are prominent, although somewhat greater regarding climate change due to substantive differences and more partisan opposition. Regarding climate change and to a lesser extent renewable energy, political divisions tends to widen with education. There also are robust age and temporal effects: younger adults more often prioritize renewable energy development, and agree with scientists on the reality of anthropogenic climate change (ACC). Across all age groups and both regional series, support for renewable energy and recognition of ACC have been gradually rising. Contrary to widespread speculation, these trends have not visibly responded to events such as the US hurricanes of 2012, 2017 or 2018. Together with age-cohort replacement and the potential for changes in age-group voting participation, however, the gradual trends suggest that public pressure for action on these issues could grow.

## 1. Introduction

The topics of climate change and renewable energy are often linked in policy discussions and scientific analysis. Mitigation of increasingly severe impacts from anthropogenic climate change (ACC) will require steep reductions in fossil fuel burning, and corresponding shifts to energy from renewable sources that produce less greenhouse gases—such as electricity generated by wind, solar or tidal power. Reduction of greenhouse gas emissions therefore becomes a key argument favoring renewable-energy development.[1] It is not the only argument, however. Renewable energy offers economic advantages including lower costs as well as new jobs, and income to producers or landowners.[2][3][4] Compared with coal or oil, it tends to generate less pollution of land, air and water. Decentralized renewable energy such as rooftop solar also promises some degree of consumer independence. These non-greenhouse arguments appeal to many of the same people concerned about climate change, but they can also reach beyond, to some who reject the reality of ACC. At the same time, the perceived impacts of large-scale energy developments such as wind farms can inspire local opposition from people who otherwise might support action on climate change.[5][6][7][8][9][10] Thus, renewable energy has potentially broader appeal, but sometimes also broader-based opposition, compared with public concern about climate change.

Scientists who study this topic express overwhelming agreement that humans are changing Earth’s climate.[11][12] Among US political leaders and public, on the other hand, partisan divisions remain wide. [13][14][15][16]. Conservatives are much less likely than moderates or liberals to agree with scientists that ACC is occurring, or that anything should be done to slow it down. The association between climate-change views and sociopolitical identity is so strong, statistically, that climate-change questions might effectively serve as proxies for political identity itself.[17] Renewable-energy opinions also correlate with sociopolitical identity, but that relationship is somewhat weaker for several reasons. Renewable-energy cost, employment or independence arguments appeal to some conservatives; and large-scale energy developments such as wind farms may stir resistance focused on local impacts, regardless of views about climate. Moreover, climate change has been most intensively targeted for opposition by conservative media and elites.[13][18][19] Appealing to politically mixed audiences, advocates for renewable energy often choose to emphasize cost, employment, income or energy-independence benefits, downplaying those related to climate.[20][21][22]

How similar or different are the social bases of support for renewable-energy development, compared with those for concern about climate change? Is public opinion shifting similarly on both topics? In a recent paper we explored these questions using data from four US survey projects—three regional and one national in scope. The nationwide survey took place in 2016, with stages just before and after the presidential elections. The three regional surveys involve places with recent and controversial wind energy developments, as described by Hamilton et al.[23] One of the regional surveys, in the North Country of northern New England, occurred in summer of 2017. The other two regional projects, in northeast Oregon and New Hampshire, each involved a series of surveys carried out over multiple years—2011 to 2015 in Oregon, and 2010 (climate) or 2012 (renewable energy) to 2017 in New Hampshire.

In this paper we elaborate earlier analyses, drawing on new data (more than 3,000 additional interviews) that extend the Oregon and New Hampshire timelines through fall 2018. We also draw on a larger set of historical data from New Hampshire, so the total now exceeds 18,000 interviews for some analyses. Extended timelines broadly confirm earlier results, while enabling new analyses including tests for public-opinion impacts from the disastrous hurricane seasons of 2017 and 2018, detailed breakdowns that find similarities in the 2018 social profiles of renewable-energy and climate views in two different regions, comparisons of trend lines for different age groups across each issue and each region, and more precise estimates of interactions as well as distinct age and trend effects.

## 2. Four survey projects

Data analyzed here come from four projects summarized in **Table 1**. For each project, trained personnel at the Survey Center of the University of New Hampshire conducted cell and landline telephone interviews with randomly-sampled participants. The nationwide POLES survey took place in two stages just before and after the 2016 presidential elections, with little difference in the main response patterns.[23] The North Country survey took place in summer 2017, interviewing residents of four rural counties in northern New England.[24][25] Two other regional projects, covering New Hampshire and northeast Oregon, each involved a series of surveys carried out with independent random samples from 2010 or 2011 to 2018. Many papers present results from the various Oregon surveys up to 2015 [26][27][28][29], and New Hampshire surveys up to 2017.[30][31] Results from the 2018 Oregon and New Hampshire surveys are described for the first time in this paper.

**Table 1:** Four survey projects.

*Polar*, *Environment*, *and Science* (POLES, nationwide). The POLES survey involved random-sample telephone interviews (cell and landline) with respondents from all US states, carried out in two stages: before the 2016 presidential elections (August, *n* = 704) and immediately afterwards (November/December, *n* = 707). Response rates in four subsamples of the POLES survey ranged from 15 to 30%, calculated by AAPOR definition 3.[32] Several papers have focused on POLES results.[33][34]
*Granite State Poll* (GSP, New Hampshire statewide). These landline and cell telephone surveys interview independent, statewide random samples of New Hampshire residents four times each year. Along with standard background and political questions, the GSP often carries items about environment or science. New Hampshire responses on environmental questions commonly fall close to national benchmarks. Several recent papers make comparisons between New Hampshire and nationwide surveys.[14][35] The GSP from April 2010 to October 2018 conducted 20,786 interviews that included our climate-change question, and from July 2012 to October 2018 conducted 7,707 with the question about renewable energy. Median response rate over this period was 21.5 percent.
*Communities and Forests in Oregon* (CAFOR, northeast Oregon). Under the CAFOR project, landline and cell telephone surveys involving independent random samples of northeast Oregon residents were conducted in four stages: September/October 2011 (*n* = 1,585 from Baker, Union and Wallowa Counties); August/October 2014 (*n* = 1,752, from the same three counties along with Crook, Grant, Umatilla and Wheeler Counties); October/November 2015 (*n* = 651, repeating the seven counties from 2014); and September 2018 (*n* = 1,097) in just the three original counties. Median response rate of the CAFOR surveys was 38 percent. For consistency, our analysis in this paper focuses on 3,782 interviews from only those counties (Baker, Union and Wallowa) that were surveyed in all four years. Voting patterns in all of these northeast Oregon counties tend to be politically conservative, and surveys find lower-than-national recognition of anthropogenic climate change.[14][23]
*North Country* (northern New England). In summer 2017, researchers with the Carsey School of Public Policy (University of New Hampshire) conducted this random-sample cell and landline telephone survey of 1,650 residents in four contiguous northern New England counties, collectively termed the North Country: Coös and Grafton Counties, New Hampshire; Essex County, Vermont; and Oxford County, Maine. Designed to assess changes in residents’ perceptions of their rural communities, the 2017 survey (response rate of 19%) replicated some questions from earlier surveys, but also included new environmental and climate items. Some results are analyzed in papers by Hamilton et al.[24][25]

With each individual survey, probability weights were calculated for adjustments toward more representative results. Following standard formulas, the weights compensate for potential bias arising from sampling design (household size; number of phones in each household; deliberate oversampling of smaller areas or subpopulations of interest) or from differential responses with respect to population age/sex distributions of the places being polled at that time. Consequently, after weighting the results should reasonably represent target populations. Effects of this weighting tend to be substantively minor. For example, support for renewable energy development among POLES respondents equals 67 percent before weighting and 72 percent after; among North Country respondents the corresponding results are 75 percent before and 78 percent after. Weighted results, which better represent regional or nationwide populations, appear in all tables and graphs of this paper.

Although research objectives varied across projects, and to a lesser extent across stages within each project, many surveys carried two standard questions asking about renewable energy and climate change. **Table 2** gives the wording of these *renew* and *climate* questions, along with codes used for modeling later. The surveys asked also about respondent background characteristics, for the most part with identical wording. One exception is that the New Hampshire surveys asked respondents for their ideological identification, here coded from –2 (extremely or fairly liberal) to +2 (extremely or fairly conservative). The Oregon surveys recorded political party identification but not ideology, so our analysis employs a simple three-party scheme from –1 (Democrat) to +1 (Republican) with these data. Political and education variables are centered at zero for use with interaction terms later. Table 2 summarizes the independent variables as well.

**Table 2:** Energy, climate change and background questions asked on multiple iterations of the New Hampshire Granite State Poll (GSP) and northeast Oregon Communities and Forests in Oregon (CAFOR) surveys over 2010 or 2011 to 2018; on two iterations of the nationwide POLES survey in 2016; and on the one-time North Country survey in 2017. Shown with codes used for logit regression analyses in Table 3. Order of response choices for *renew* and *climate* were rotated across interviews.

*Renew*—Which do you think should be a higher priority for the future of this country, increased exploration and drilling for oil, or increased use of renewable energy such as [tidal,] wind or solar? Reference to tidal energy occurred only in the New Hampshire surveys, although tests indicate that this word made no difference. Increased use of renewable energy such as [tidal,] wind or solar (1) Increased exploration and drilling for oil (0) don’t know/no answer (0)
*Climate*—Which of the following three statements do you think is more accurate? Climate change is happening now, caused mainly by human activities (1) Climate change is happening now, but caused mainly by natural forces (0) Climate change is not happening now (0) Don’t know/no answer (0)
*Age*—Respondent’s age in years
*Sex*—Male (0) or female (1)
*Education*—High school or less (–1), some college or technical school (0), college graduate (1), or postgraduate (2)
*Ideology* (New Hampshire GSP surveys)—Extremely or fairly liberal (–2), somewhat or leaning liberal (–1), moderate not leaning (0), somewhat or leaning conservative (1), extremely or fairly conservative (2)
*Party* (Oregon CAFOR surveys)—Democrat (–1), Independent (0), Republican (1)
*Year*—Year of survey, from 2010 (New Hampshire) or 2011 (Oregon) to 2018

**Fig 1:**
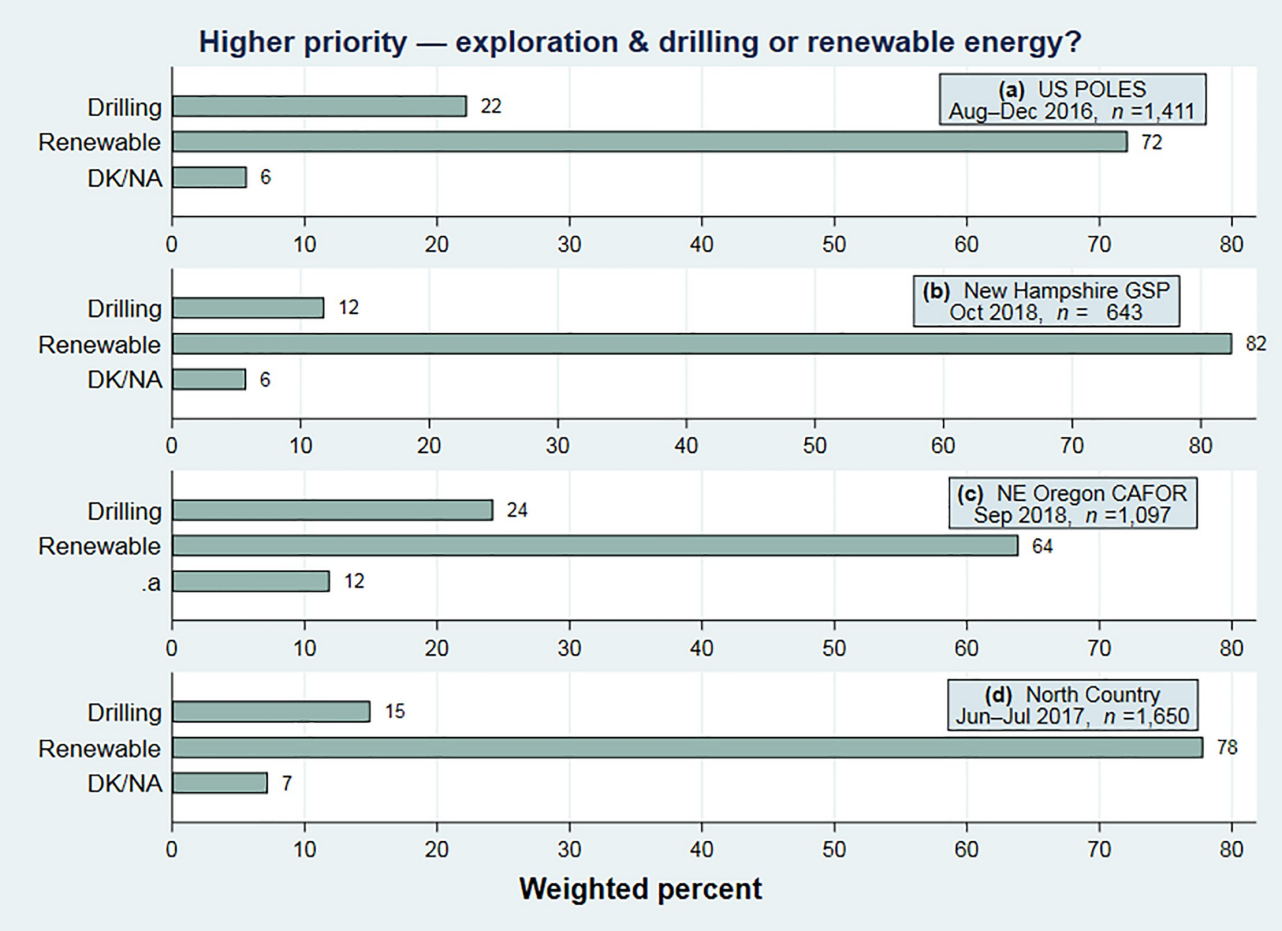
Should increased exploration and drilling for oil, or increased use of renewable energy such as wind or solar, be a higher priority for the future of this country? Results from the most recent years of four survey projects.

In the New Hampshire and Oregon series, as will be seen, response patterns on the renewable energy and climate questions both changed over time. **Fig 1** charts responses to renewable-energy (*renew*) responses from the most recent year of each project: 2016 for the US POLES survey, 2017 for North Country, or 2018 for northeast Oregon and New Hampshire. Large majorities of the respondents on each survey, between 64 and 82 percent, consider increased use of renewable energy to be a higher priority. The lowest number, 64 percent favoring renewable energy, represents northeast Oregon—politically a very conservative region, where 67 to 73 percent of the voters in each county supported Trump in 2016. Despite that region’s general conservativism, support for renewable energy is only 8 points lower in northeast Oregon than it is nationwide (64 versus 72).

The two highest values in Fig 1, 78 or 82 percent favoring renewable energy, represent recent surveys in comparatively moderate and mixed regions: the North Country (counties voting from 38 to 57 percent for Trump) or New Hampshire (counties voting from 38 to 56 percent for Trump). The *renew* response “Increased exploration and drilling for oil” echoes a campaign slogan—“Drill, baby, drill”—popularized by Republicans during the 2008 US presidential race, then revived in 2012 and 2016. Despite substantial Republican presence in every region assessed, this response was chosen by less than a quarter of the respondents.

**Fig 2:**
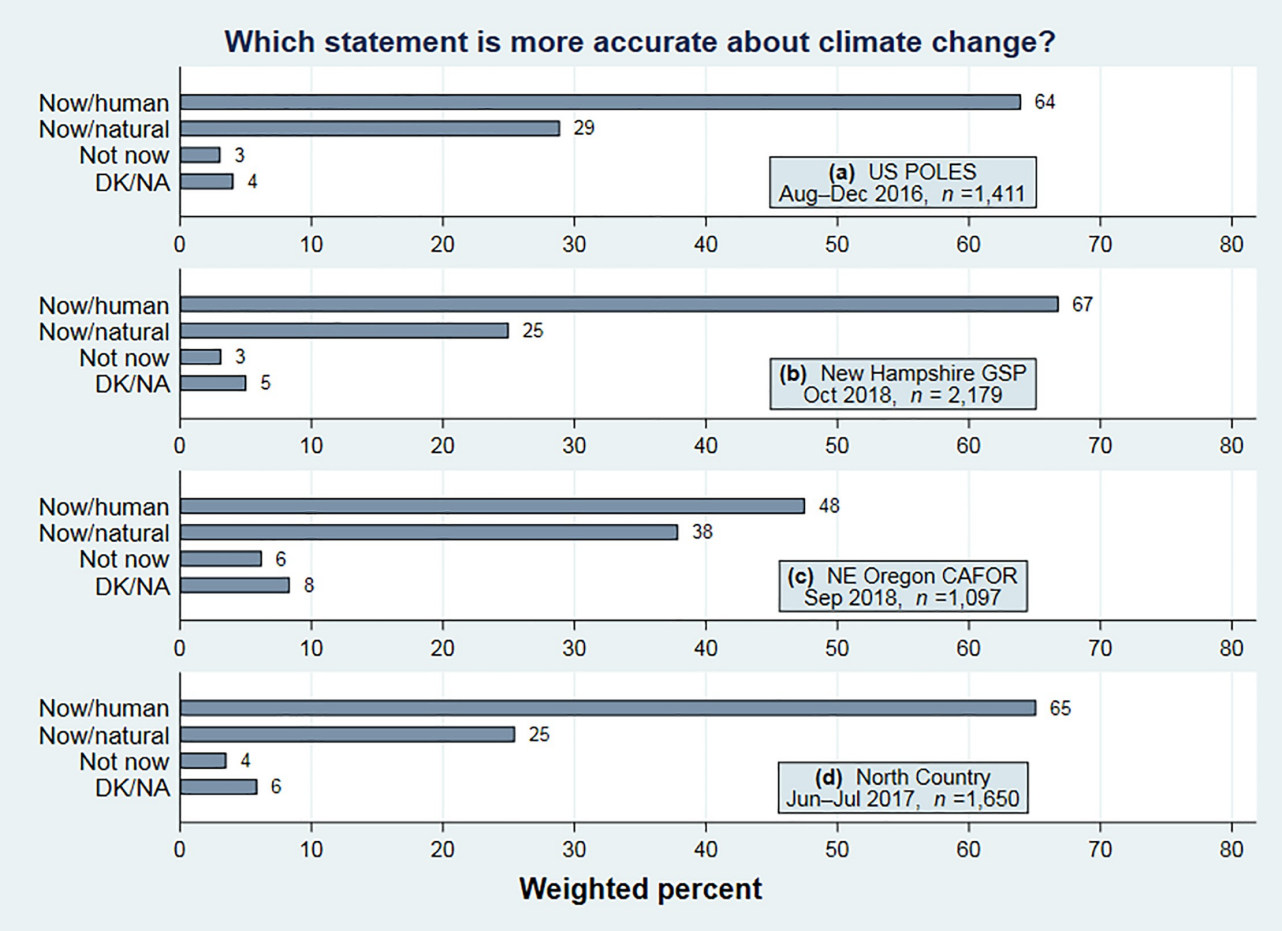
Is climate change happening now, caused mainly by human activities? Is it happening now, but caused mainly by natural forces? Or is climate change not happening now? Results from the most recent years of four survey projects.

**Fig 2** charts response to the climate-change question (*climate*) in parallel fashion. Again, only the most recent years of each project are shown; in New Hampshire the *climate* question was asked more frequently than *renew*, so we have more observations. US, New Hampshire and North Country results are quite similar: 64 to 67 percent agreeing with the scientific consensus that climate change is happening now, caused mainly by human activities. In contrast, fewer than half of the northeast Oregon respondents (48 percent) accept this consensus. The gap between northeast Oregon and US views on this item is 16 points (48 vs. 64), double what we saw on renewable energy. The Oregon respondents are comparatively more likely than nationwide or other regional respondents to think climate is changing mainly for natural reasons (38 percent) or even that it is not changing (6 percent), despite summer warming that has worsened the wildfires affecting their region.[28][29]

Comparisons between renewable-energy and climate-change views in Figs 1 and 2 suggest that renewable energy development is viewed favorably by many people who do not believe that human activities are changing the climate. Moreover, the aggregate results imply that renewable energy views correlate less strongly with politics. The next section tests this inference directly, and explores how other characteristics correlate with these views.

## 3. Social bases of energy and climate opinions

Decades of survey research on the social bases of environmental concern has established robust patterns with regard to respondent age, sex, education and politics. Concern about environmental problems, across many different topics, tends to be higher among younger, female and better educated respondents. In some data one or more of these effects may be relatively weak or not significant, but they almost always point in the same direction. The most consistently dominant predictors of environmental concern, however, are ideology or political identity: conservatives less often view environmental problems as serious.

**Fig 3:**
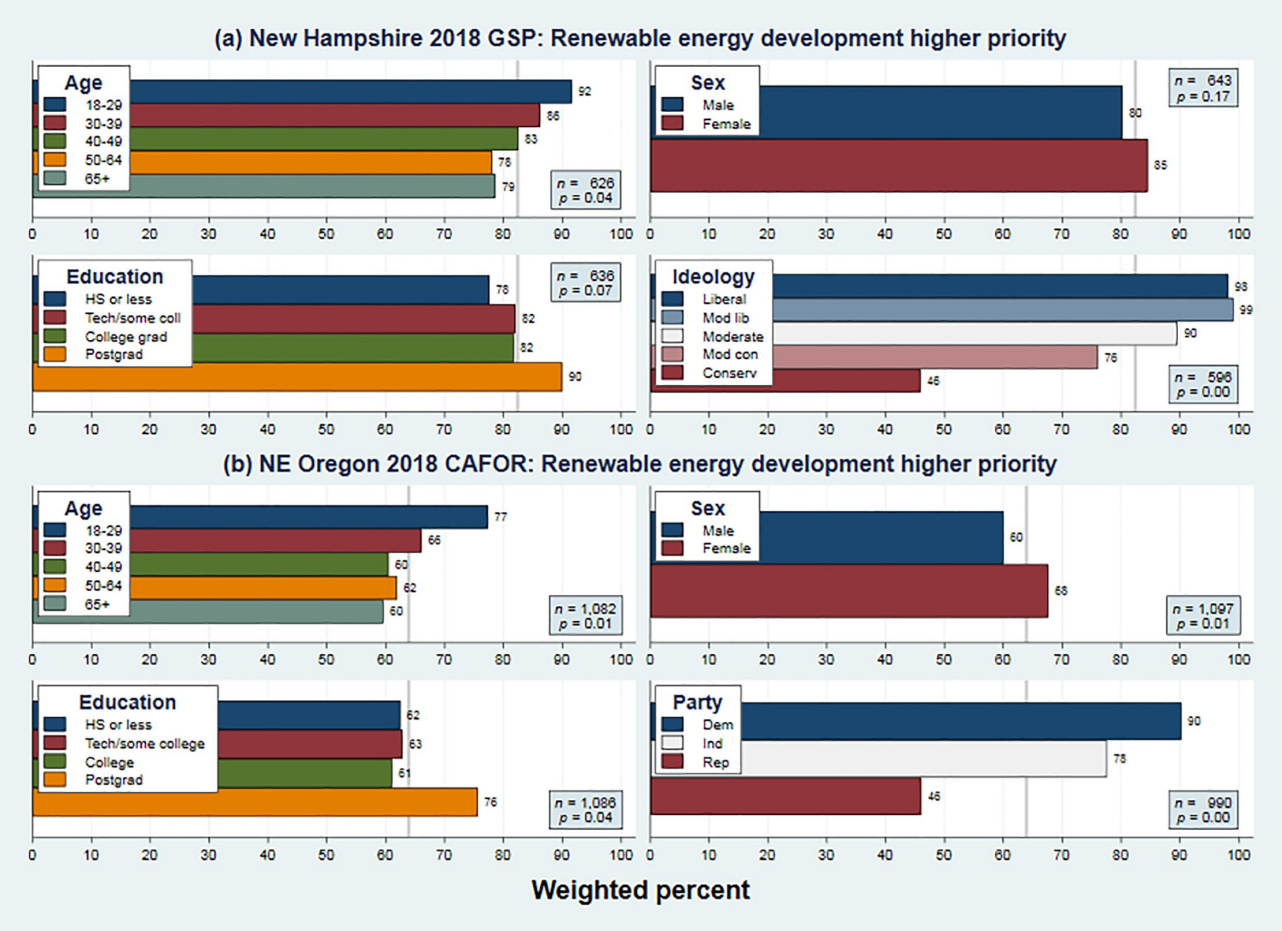
Weighted percentages for “renewable energy higher priority” broken down by respondent age, sex, education and ideology or party on surveys conducted in 2018: (a) statewide New Hampshire, and (b) northeast Oregon.

**Fig 4:**
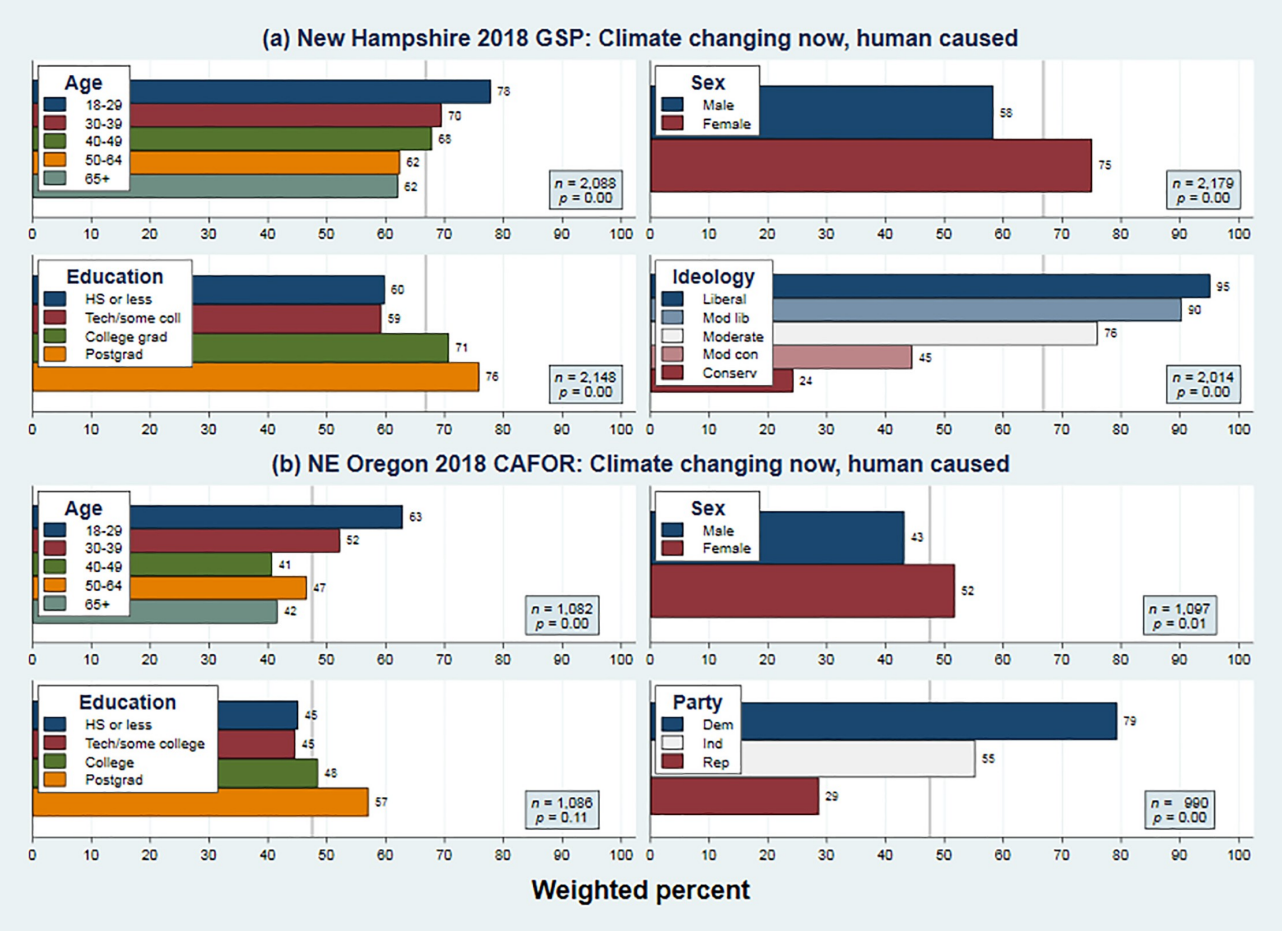
Weighted percentages for “climate change happening now, caused mainly by human activities” broken down by respondent age, sex, education and ideology or party on surveys conducted in 2018: (a) statewide New Hampshire, and (b) northeast Oregon.

**Fig 3** and **Fig 4** show that these propositions apply to views on renewable energy and climate change. In each figure the (a) panels depicts 2018 New Hampshire data, and the (b) panels the 2018 Oregon data. Generally similar results also occurred in the North Country and nationwide surveys, and in earlier years from the New Hampshire and Oregon projects (not shown). Younger, female and better educated respondents more often prioritize renewable energy, and more often agree that humans are changing Earth’s climate. Across each of these four panels, ideological or political indicators have by far the strongest effects, with liberal-conservative or Democrat-Republican gaps of 53 or 44 points on renewable energy, and 71 or 50 points on climate. Age differences are less strong but also consistent, with young versus old gaps of 13 or 17 points on renewable energy, and 16 or 22 points on climate.

Figs 3 and 4 confirm that in 2018, renewable-energy and climate-change views have similar demographic predictors, which resemble those known for many other environment-related topics. Both exhibit steep political gradients, and milder but also significant age gradients. In detail, this four-way comparison reveals something else. Although the ideological or partisan gaps regarding renewable energy on both surveys are wide, they are less wide than the corresponding gaps regarding climate change. So renewable energy opinions do strongly correlate with politics, but not as strongly as climate-change opinions—as inferred indirectly from regional comparisons in Figs 1 and 2.

## 4. Trends over time

**Fig 5:**
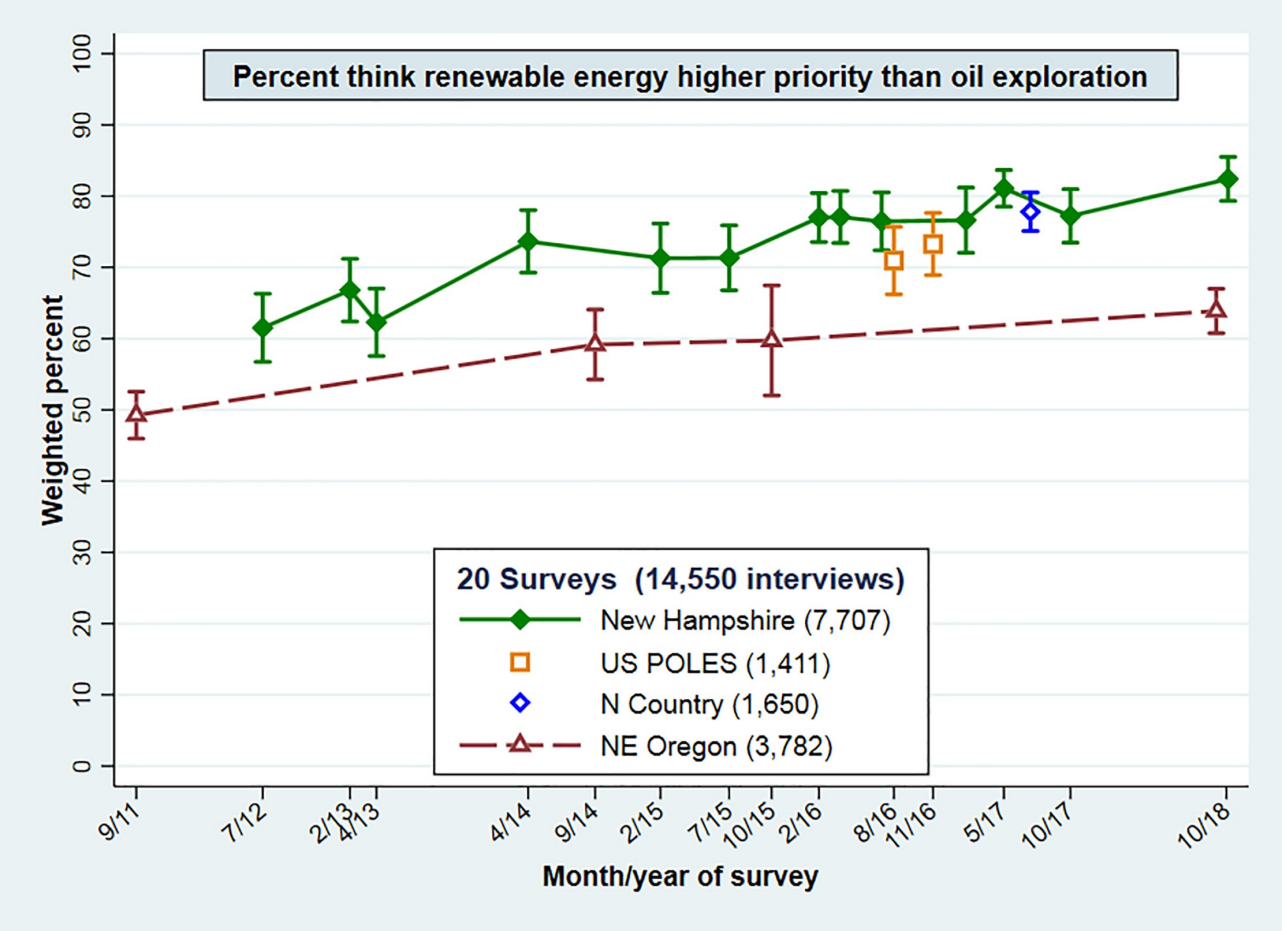
Weighted percentages and 95 percent confidence intervals for “renewable energy higher priority” on two nationwide and 18 regional (New Hampshire, North Country or northeast Oregon) surveys. Combined *n* = 14,550.

From 2012 through fall of 2018, 13 New Hampshire surveys with a combined total of 7,707 interviews carried the renewable-energy question, as did four northeast Oregon surveys (2011–2018) with 3,782 interviews. **Fig 5** tracks these regional-survey results, along with results on the same question from the POLES and North Country surveys. Unlike Figs 1–4 which depict only the most recent years of each project, Fig 5 and subsequent graphs employ all available data for the analysis shown. The number of observations fluctuates, because some questions were not asked on some surveys. The upper line in Fig 5, drifting up about 21 points, tracks the percentage of New Hampshire respondents who prioritize renewable energy. The lower line shows an upward drift of about 14 points among northeast Oregon respondents. Nationwide results from the POLES surveys (1,411 interviews) appear slightly lower than contemporary New Hampshire results; North Country results (1,650 interviews) match New Hampshire almost exactly. Error bars depict the 95 percent confidence intervals for each survey. We see minor survey-to-survey variations, within the range of sampling error, but the main visual impression is how stable percentages appear, from one survey to the next. Their short-term stability reflects use of consistent sampling and interview methods, repeating a straightforward question. An earlier paper based on Oregon data through 2015 and New Hampshire through 2017 observed similar upward trends,[23] which are now seen continuing in these longer time series.

**Fig 6:**
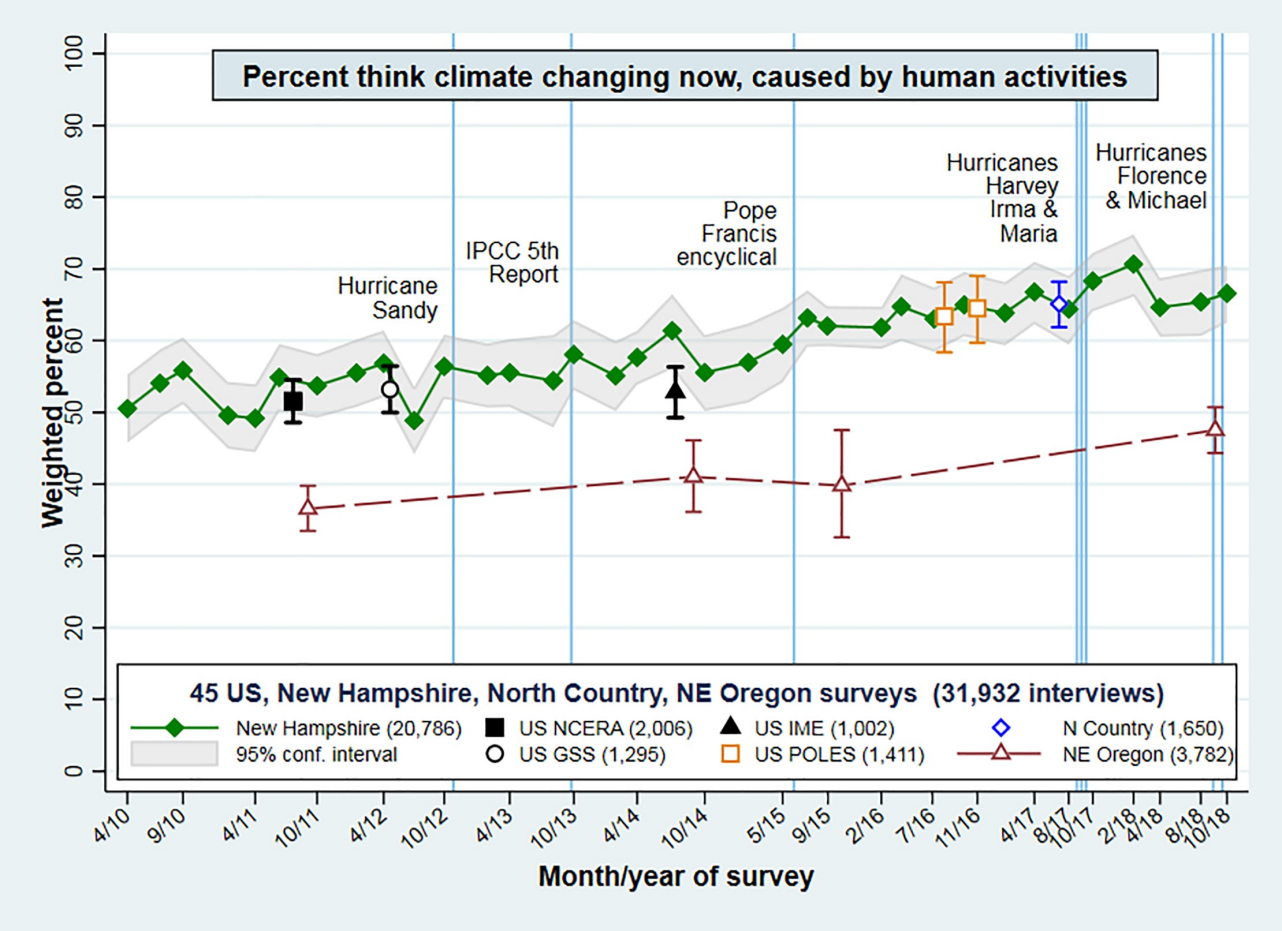
Weighted percentages and 95 percent confidence intervals for “climate change happening now, caused mainly by human activities” on five nationwide and 40 regional surveys. Combined *n* = 31,932.

We have more data on the climate-change question, which was asked on the same POLES, northeast Oregon and North Country surveys, but also on other nationwide surveys in 2011, 2012 and 2014, along with 35 New Hampshire surveys 2010–2018. **Fig 6** tracks these regional and national results. On climate change, New Hampshire public opinion is never far from national opinion, and might be viewed as a reasonable proxy. Hamilton et al. describe the older national surveys in more detail, and tracked New Hampshire results through spring of 2015.[14] Fig 6 updates that analysis with data from POLES 2016, North Country 2017, and New Hampshire and Oregon through 2018. This graph also notes some major events that were widely proposed, at the time, to potentially shift public opinion on climate change. The most recent such events in this timeline are the disastrous US hurricane seasons of 2017 (Harvey, Irma and Maria) and 2018 (Florence and Michael). But these hurricane seasons, like the earlier events of Hurricane Sandy (2012), the IPCC 5^th^ report (2013), and Pope Francis’ encyclical on climate change (2015), produce no visible change. Together with science communication, however, such events might contribute to the cumulative rise.[35]

Both the renewable-energy results in Fig 5 and the climate-change results in Fig 6 exhibit minor survey-to-survey fluctuations, within sampling error bars. Overall, however, they show substantial consistency around upward trends. Northeast Oregon acceptance of ACC remains well below national or northeastern levels, but drifts similarly upward over this period. Multivariate analysis will later establish that the trends of both Oregon and New Hampshire series in Figs 5 and 6 are statistically significant.

**Fig 7:**
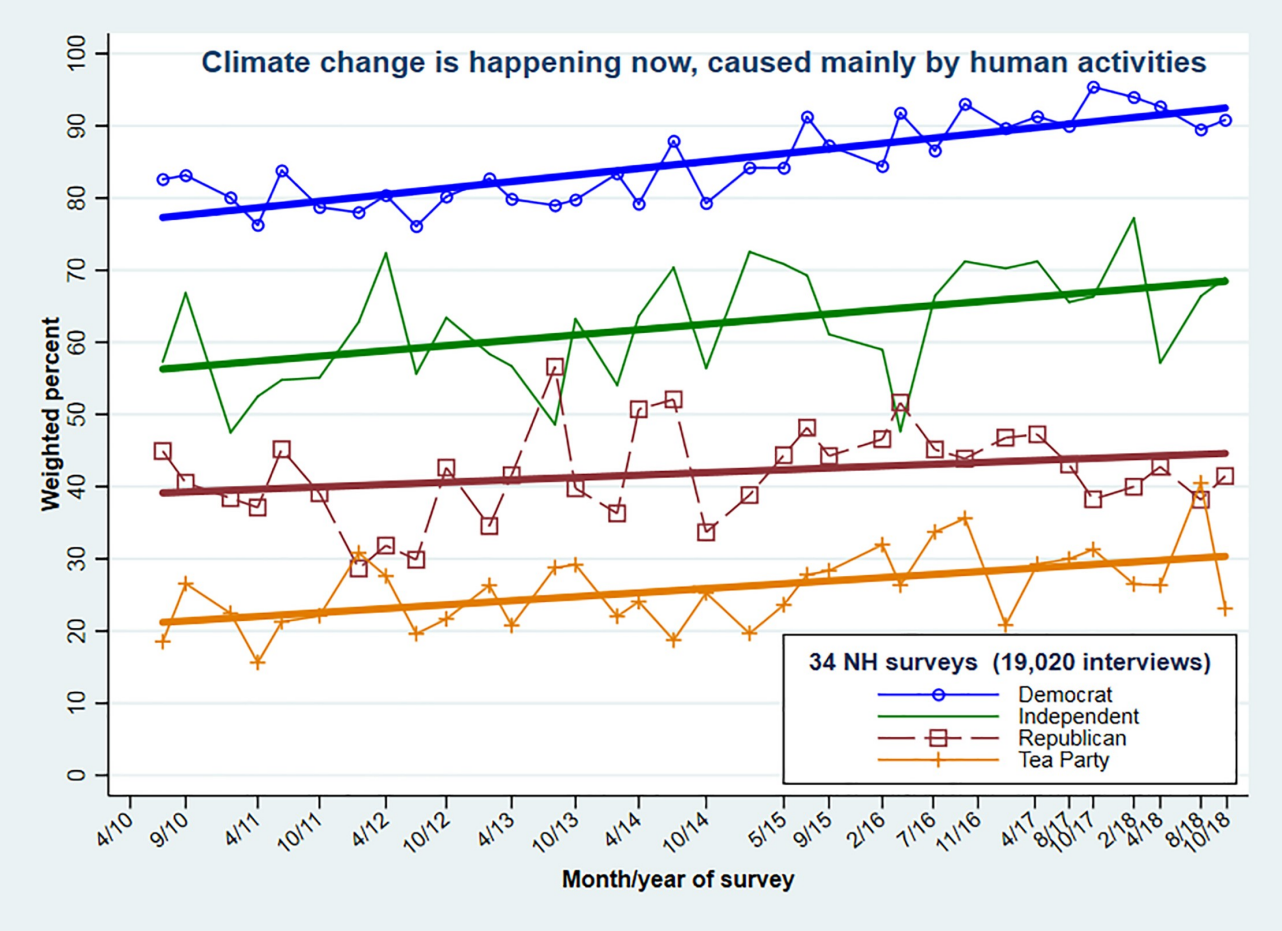
Weighted percentages “climate change happening now, caused mainly by human activities,” by respondent political party on 34 New Hampshire surveys. Combined *n* = 19,020.

Replication of the overall percentages seen in Figs 5 and 6 extend to more detailed analyses as well, although sample-to-sample variation increases as we look at smaller subsamples. For example, **Fig 7** tracks climate-change percentages separately for each political party, across 34 New Hampshire surveys. The four parties exhibit roughly parallel upward trends. Separation into five levels of ideology from liberal to conservative (not shown) paints a similar picture as well.

**Fig 8:**
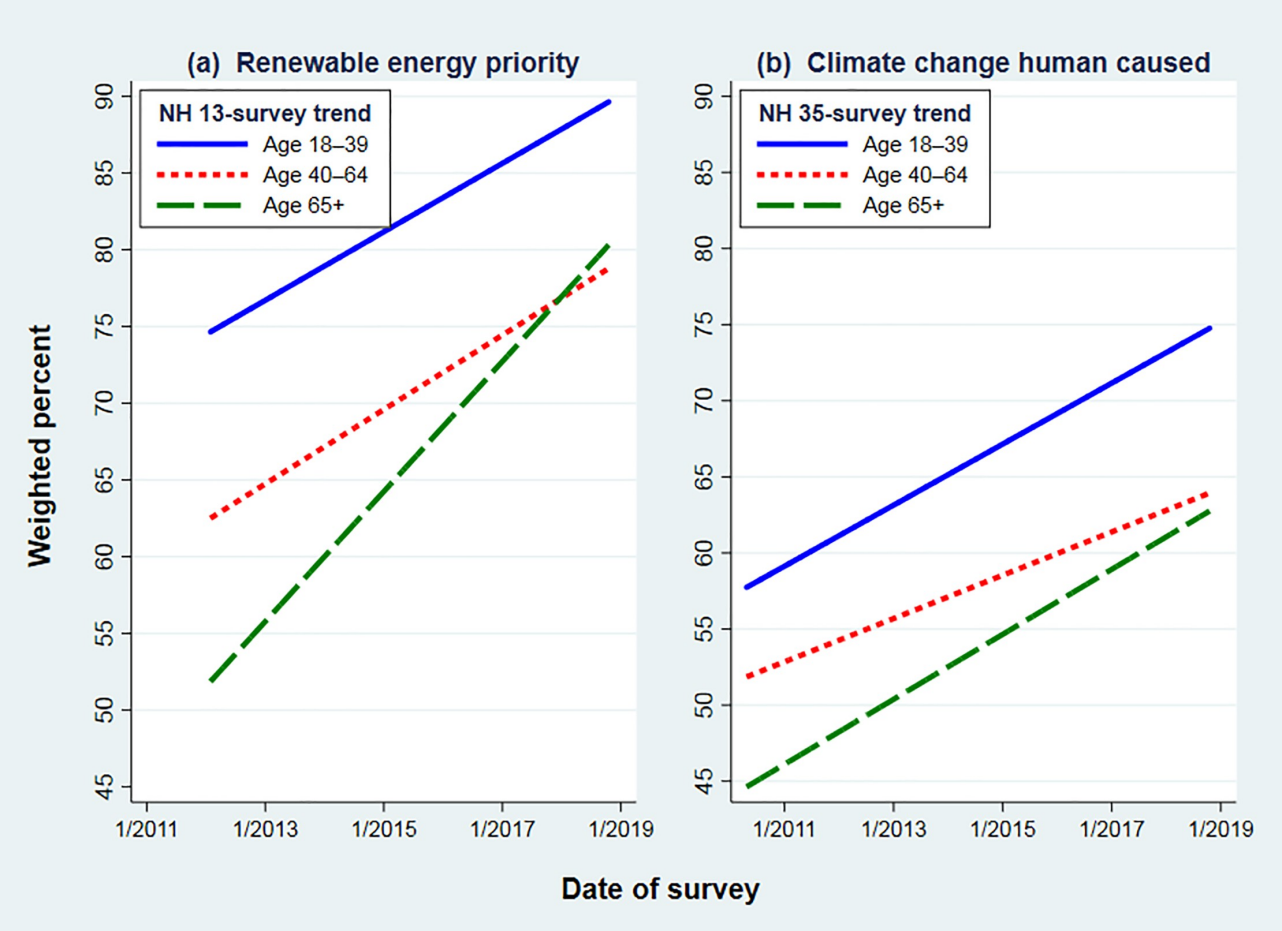
Linear (ordinary least squares) trends by age group on statewide New Hampshire surveys for (a) “renewable energy higher priority” 2012–2018, or (b) “climate change happening now, caused mainly by human activities” 2010–2018.

**Fig 8** shows a different breakdown, graphing New Hampshire renewable energy (13 surveys) and climate change (35 surveys) trends separately by age group. Linear trends rather than separate data points are depicted because survey-to-survey variations in mall subsamples otherwise make the graphs hard to read. The pro-environmental or scientific positions within each age group nevertheless show clear upward trends. Millennials, roughly age 18 to 39, stand apart at the top of each panel: they are consistently more likely than older groups to favor renewable energy, or to think that humans are changing the climate. Moreover, support among Millennials has been rising steadily: now past 90 percent on energy and 75 percent on climate.

Respondents 65 and older start out this period below anyone else, but their trends climb upward as well, reaching 75 percent on energy and 60 percent on climate. Toward the end of this period, in both panels there appears to be some convergence of old and middle-aged respondents. Middle-aged percentages start halfway between young and old, but rise less steeply than the others.

**Fig 9:**
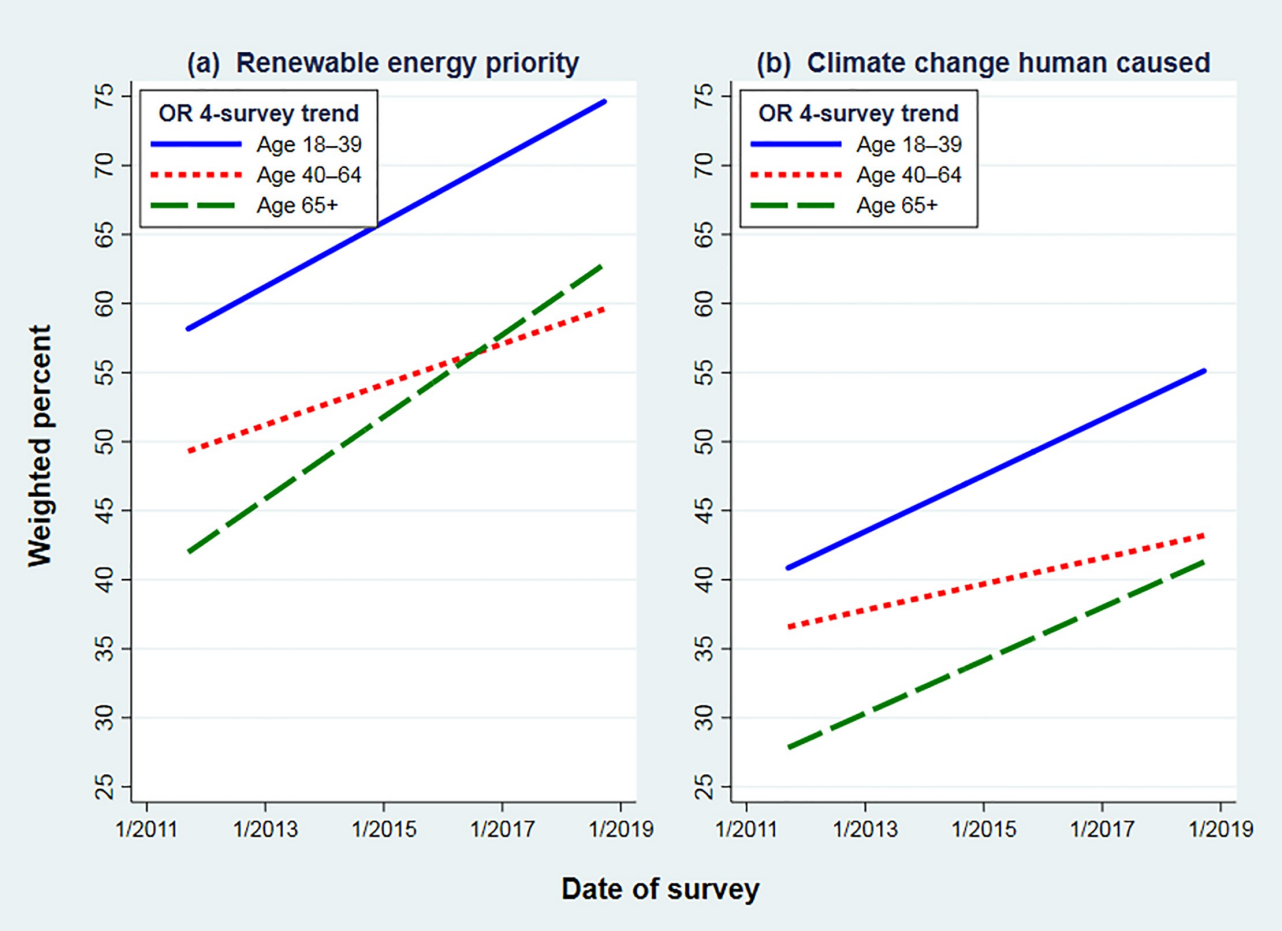
Linear (ordinary least squares) trends by age group on northeast Oregon surveys 2011–2018, for (a) “renewable energy higher priority”, or (b) “climate change happening now, caused mainly by human activities”.

In **Fig 9** we calculate the corresponding age-group trends from Oregon data. The wider gap between renewable-energy and climate views in this region is obvious from the different height of lines in Fig 9A and 9B. Renewable energy support also rises more steeply than ACC acceptance. A tertiary detail, in which Oregon results echo New Hampshire, is the apparent convergence of middle-aged and older views, as middle-aged views rise less steeply.

## 5. Effects of age, education and politics

Figs 3 and 4 chart bivariate relationships between renewable-energy or climate responses and four background factors that often predict environment-related views. Figs 5–9 track the upward drift in these views over time. **Table 3** tests these background and timing factors together. The table gives odds ratios from four weighted logistic regression models with respondent characteristics and yearly trend as predictors. Each model draws on all available data—including more historical survey data as well as the most recent, so estimation samples include about 1,000 additional interviews for both Oregon models, and 700 (*renew*) to 14,000 (*climate*) additional interviews for New Hampshire, compared with previously published analyses.[23] Consequently, parameter estimates here are more precise.

**Table 3:** Respondent characteristics and survey timing as predictors of high priority for renewable energy (*renew*), or think climate change is happening now, caused mainly by humans (*climate*). Values shown are odds ratios (*e*^*b*^) from probability-weighted logit regression with either New Hampshire GSP or NE Oregon CAFOR survey datasets (3 original counties), pooled over all available years.

	Surveys and Dependent Variables				
	New Hampshire GSP		NE Oregon CAFOR	
Predictor	*1*. *Renew*	*2*. *Climate*	*3*. *Renew*	*4*. *Climate*
*Age*	0.983***	0.988***	0.982***	0.982***
*Sex* (female)	0.990	1.373***	1.069	1.079
*Education*	1.124**	1.203***	1.173**	1.225***
*Ideology*	0.485***	0.501***	…	…
*Education×ideology*	0.876***	0.827***	…	…
*Party*	…	…	0.348***	0.368***
*Education×party*	…	…	0.931	0.778***
*Year*	1.179***	1.070***	1.119***	1.102***
estimation sample	6,904	18,610	3,333	3,333
*F* statistic	133.58***	430.89***	58.94***	59.87***

* *p* < 0.05

** *p* < 0.01

*** *p* < 0.001 (Wald tests)

Odds ratios significantly above 1.0 for *year*, across all four models in Table 3, confirm the upward drift of support for renewable energy and acceptance of ACC in both New Hampshire and Oregon data (*p* < 0.001). We also see odds ratios below 1.0 for *age* across all four models (*p* < 0.001). That is, older respondents in both New Hampshire and Oregon are less inclined to prioritize renewable energy, and also less inclined to believe that humans are changing the climate. (Note that *age* is entered as a measurement variable—simply, age in years—for the models of Table 3, although we grouped age for readability in the graphs.) The *year* and *age* effects agree with simpler results visualized in Figs 8 and 9: clear ordering and wide separation of response preferences by *age regardless of trend*, but also *upward trends regardless of age*.

Women accept the reality of ACC at higher rates than men do in our New Hampshire data, but other sex differences in Table 3 are not significant. *Education*, like *age* and *year*, affects responses across all four models. Because *education* appears also in interactions with *ideology* or *party*, these main effects from *education* (with odds ratios significantly above 1.0) represent the positive influence of education among political moderates (*ideology* = 0) or Independents (*party* = 0). Moderates or Independents who have college educations are more likely than their peers to support renewable energy, and to think that ACC is real. By similar reasoning, the main effects of *ideology* and *party* represent the effects of these characteristics among respondents who completed technical school or some college (*education* = 0).

**Fig 10:**
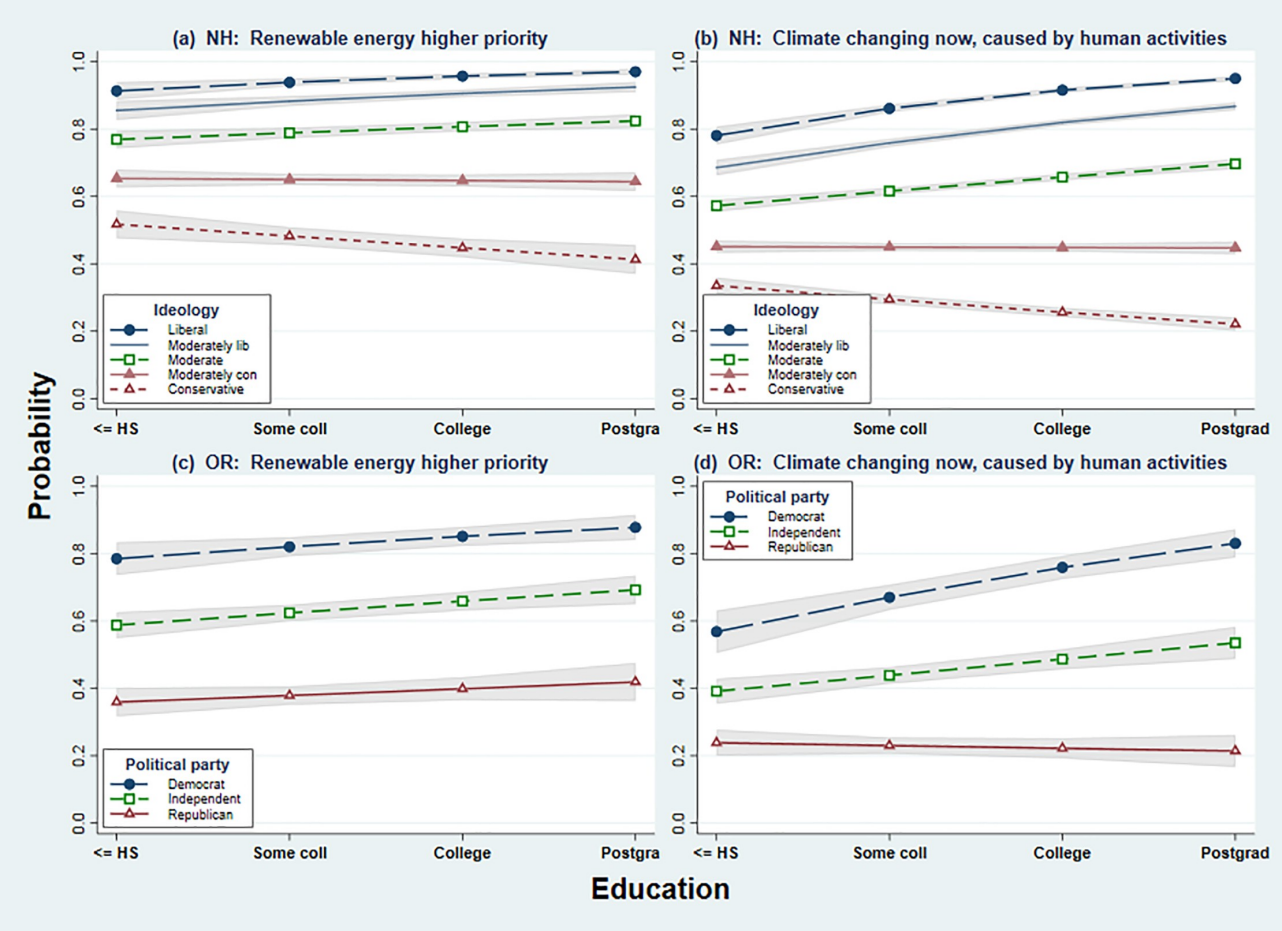
Probability of prioritizing renewable energy, or recognizing the reality of anthropogenic climate change, by education and ideology (New Hampshire) or political party (northeast Oregon). Adjusted margins plots calculated from models of Table 3.

The effects of education vary, however, depending on political identity. The *education×ideology* or *education×party* interactions in Table 3, significant (*p* < 0.001) in all but model 3, replicate a result that has been widely noticed in other studies of environment or science-related topics: the partisan spread on many issues widens with education, so better-educated partisans stand the farthest apart. **Fig 10** visualizes these effects through adjusted margins plots, calculated from models 1–4 in Table 3. In both New Hampshire (top) and Oregon (bottom) data, this interaction is strongest regarding climate change. A similar though weaker effect can be seen in the New Hampshire responses on renewable energy, as well. These findings offer more precise estimates that confirm general conclusions of Hamilton et al.[23]

Each of the panels in Fig 10 shows that education has a positive effect on renewable-energy support, or acceptance of ACC, among liberals and moderates (or among Democrats and Independents). Education has a negative effect, however, on ACC acceptance among the most conservative, or among Republicans, in both New Hampshire and Northeast Oregon (Fig 10B and 10D). Education similarly has a negative effect on renewable energy support in New Hampshire: better-educated conservatives are less inclined to support it. Education effects on renewable-energy support in northeast Oregon are very weak.

The Fig 10 results join a substantial list of other studies and datasets where education×politics or similar interaction effects are reported.[14][17][30][36][37][38][39][40][41][42][43] The “right-facing megaphone” shape of polarization widening with education, as seen in Fig 10A, 10B and 10D, reflects a pervasive reality of current US politics.

## 6. Discussion

Strimling et al., analyzing responses to “moral issue” questions on the US General Social Survey over the past 40 years, find a common theoretical explanation that fits divergent trends.[44] Public opinion has become more favorable toward positions whose moral foundations involve fairness and harm, values favored by liberals and conservatives alike. Opinion has not moved as much on issues whose moral foundations involve authority, loyalty or purity, values favored mainly by conservatives. This moral-issue analysis does not address environmental, economic or policy-effectiveness issues, which have central importance for energy and climate. Fairness and harm values have also been prominent in discourse on energy and climate, however, so the Strimling et al. moral dynamic might contribute to the upward trends observed in our study as well.

The strong effect sociopolitical identity exerts on US public opinion about climate change is well known, and confirmed once again here. Despite divergence in the rationales regarding climate and renewable-energy development, we see that sociopolitical identity has major impacts on energy views too—but those impacts are somewhat weaker. The education×politics interaction effect, describing a partisan gap that widens with education, appears less pronounced on renewable energy as well. These muted partisan divisions indicate that renewable-energy views are less closely bound to sociopolitical identity, in public opinion as in elite communication such as the campaigns against climate science.[13][18][19] Substantively, some conservatives who reject the scientific consensus on climate change nevertheless embrace renewable energy on other grounds, such as employment or income benefits, or contributions to energy independence. At the same time, some moderates or liberals, who agree in principle on the need for greenhouse gas reductions, oppose developments such as wind farms that will have local impacts. Expansion of low-carbon renewable energy remains an essential step for climate-change mitigation, but might be motivated by different arguments in some contexts, and while also requiring more local efforts at public engagement—as renewable-energy proponents tend to be well aware.

Our results include several new findings as well: no visible impacts on public opinion from five disastrous hurricanes that struck the US in just two seasons, 2017 and 2018; and detailed correspondences between the social bases of renewable energy support, and of ACC acceptance, across two very different US regions. Those results highlight stability, but other findings hint at future change. First, there are consistent age effects. In the most recent surveys more than 90 percent of New Hampshire respondents age 18 to 29 favor renewable energy, as do 77 percent in northeast Oregon. Those fractions are 13 or 17 points higher than they are among people over age 65. The generation gap is even wider (16 or 21 points) regarding climate change. Age has significant positive effects on both climate and energy views even after controlling for sex, education, political identification and year of survey. Assuming no change in people’s individual views, gradual cohort replacement could raise public acceptance of renewable energy and other climate-change mitigation steps. If voter participation rates among young adults rise, as some observers expect, the balance might shift more quickly.

A separate result is that, over the period of observation, support for renewable energy and acceptance of anthropogenic climate change have been gradually rising. For each series in both datasets, the increase exceeds 10 percentage points, with renewable energy climbing at slightly faster rates than climate. It is worth noting that these trends are established in models that already account for respondent age, so they do not reflect possible cohort shifts noted above. By the same token, the age effects estimated in those models are independent of overall trends. We see distinct age and temporal patterns in terms of separate upward trends within each age group. These trends add further reason, besides the certainty of cohort replacement and the possibility of more voting by young adults, to think that public support for action on these issues will grow.

## 7. Conclusion

Earlier survey research on renewable energy and climate change is extended here using data that include 3,000 new survey interviews, through fall 2018, for two regional time series (New Hampshire and northeast Oregon, benchmarked by nationwide surveys). The extended timelines broadly confirm earlier trends while filling in new details. We see no short-term impacts on public opinion from events such as the disastrous US hurricanes of 2012, 2017 or 2018. Instead, there have been gradual upward trends in acceptance of anthropogenic climate change, and in support for renewable-energy development. These overall trends are repeated within age groups of our regional series. Climate and renewable-energy views in both regions have similar social profiles, including generational differences, and political divisions that are narrower on renewable energy than climate. Political divisions tend to widen with respondent’s education, an interaction effect noticed previously but here confirmed with very large samples (up to 18,000 interviews) yielding more precise parameter estimates. Multivariate analysis establishes significant age effects net of trends, and trend effects net of age—both pointing toward the direction of future shifts in public opinion on these two interlinked topics.

## Supporting information

S1 Dataset Renewable energy support on 20 surveys (Stata 15 format). (DTA)

S2 Dataset Weighted percentages & conf intervals for time series (Stata 15 format). (DTA)

S3 Dataset New Hampshire climate change views by political party (Stata 15 format). (DTA)

S4 Dataset NH views on climate & renewable energy, by age group (Stata 15 format). (DTA)

S5 Dataset NE OR views on climate & renewable energy, by age group (Stata 15 format). (DTA)

S6 Dataset North Country views on climate & renewable energy (Stata 15 format). (DTA)

S7 Dataset NH views on climate & renewable energy (Stata 15 format). (DTA)

S8 Dataset POLES US survey views on climate & renewable energy (Stata 15 format). (DTA)

S9 Dataset NE Oregon views on climate & renewable energy (Stata 15 format). (DTA)

S1 File Do-file containing Stata commands to draw Figs 1–10 and perform statistical calculations for Table 3 (plain text format, executes in Stata 15 with catplot installed). (DO)
